# A Novel growth guidance system for early onset scoliosis: a preliminary in vitro study

**DOI:** 10.1186/s13018-024-04720-0

**Published:** 2024-04-24

**Authors:** You Du, Yanyan Bian, Yiwei Zhao, Yang Yang, Guanfeng Lin, Bingtai Han, Haoran Zhang, Chenkai Li, Xiaohan Ye, Zhiyi Li, Jianguo Zhang, Shengru Wang

**Affiliations:** Department of Orthopedic Surgery, Peking Union Medical College, Peking Union Medical College Hospital, Chinese Academy of Medical Sciences, 1st Shuai Fu Yuan, Dongcheng District, Beijing, 100730 China

**Keywords:** Early-onset scoliosis, Shilla system, Growth guidance system, In vitro experiments

## Abstract

**Purpose:**

The purpose of the study was to describe a novel growth guidance system, which can avoid metal debris and reduce the sliding friction forces, and test the durability and glidability of the system by in vitro test.

**Method:**

Two major modifications were made to the traditional Shilla system, including the use of ultra-high molecular weight polyethylene (UHMWPE) gaskets to avoid direct contact between the screw and rod, and polishing the surface of the sliding part of the rod. We tested the durability of the system by a fatigue test, which the samples were test on the MTS system for a 10 million cycle of a constant displacement. Pre and post-testing involved weighing the UHMWPE gaskets and observing the wear conditions. The sliding ability were measured by a sliding displacement test. The maximum sliding displacement of the system was measured after a 300 cycles of dynamic compressive loads in a sinusoidal waveform.

**Results:**

After the fatigue test, all the UHMWPE gaskets samples showed some of the fretting on the edge of the inner sides, but its still isolated and avoided the friction between the screws and rods. There was no production of metallic fretting around the sliding screws and rods. The average wear mass of the UHMWPE gaskets was 0.002 ± 0.001 g, less than 1.7% of the original mass. In the sliding test, the novel growth guidance system demonstrated the best sliding ability, with an average maximum sliding distance(AMSD) of 35.75 ± 5.73 mm, significantly better than the group of the traditional Shilla technique(AMSD 3.65 ± 0.46 mm, *P* < 0.0001).

**Conclusion:**

In conclusion, we modified the Shilla technique and designed a novel growth guidance system by changing the friction interface of sliding screw and rod, which may significantly reduce the metallic debris and promote spine growth. The fatigue test and sliding dislocation test demonstrated the better durability and glidability of the system. An in vivo animal experiment should be performed to further verify the system.

## Introduction

The treatment of early onset scoliosis (EOS) is a complex matter. While braces and casts may be effective in controlling and preventing curve progression in smaller curves, larger curves may require surgery. It is important to note that the primary goals of EOS surgery are to correct the curvature and preserve the growth potential of the spine. Currently, there are three main categories of EOS surgery techniques: distraction-based techniques, growth guidance techniques, and hybrid techniques [1]. The representative of distraction-based technique is traditional growing rods, which have the strong power to correct the curvature and preserve the growth potential [2]. However, regularly scheduled distraction surgery under general anesthesia, poor control of apical curvature, and the high risk of implant failure are the main drawbacks of the traditional growing rods [3, 4]. While magnetically controlled growing rods may avoid repeated surgeries under general anesthesia, they still have some disadvantages, including the high risk of implant failure, limited correction ability, and inability to be applied to severe EOS patients [5].

McCarthy initially invented the Shilla technique, which is the most widely used growth guidance technique [6]. The Shilla system was initially tested in goats to prove the ability to preserve the growth potential of the spine. The concept of the Shilla system is guiding spinal coronal alignment into a straighter alignment and utilizing the inherent growth potential of children’s vertebral column to allow the spine to grow. The core part of Shilla system is Shilla screws or sliding screws, which is a poly-axial pedicle screw with a locking cap that can only lock the top of the screw but capture the rod, allowing the screw can slide with the rod in a longitudinal direction. At the apex of scoliosis, traditional fixed-head pedicle screws are placed, and multiple levels of osteotomies are performed, to correct the curve maximally. The Shilla screws are placed at the two ends of the curve through the muscle layer, allowing the spine to grow and maintain the coronal and sagittal alignments.

According to the clinical reports from McCarthy, the Shilla system has demonstrated the abilities to correct curvature, allow the spine and lung cavity to grow, and significantly reduce the number of surgeries compared to the traditional growing rods [7, 8]. However, the increasing use of the Shilla system in EOS patients has raised concerns among surgeons about its weaker ability to promote growth compared to traditional growing rods [9, 10], as well as the potential adverse effects caused by the metal debris created by the friction of Shilla screws and rods in vivo [11, 12].

In order to reduce the metal debris and improve the sliding ability, we modified the traditional Shilla system and named it as the novel growth guidance system. Our design was granted an utility patent in People Republic of China(CN202121171449.6). In this study, we described the design of the novel growth guidance system, and reported the preliminary in vitro experiments results.

## Material and method

### Design of the novel growth guidance system

**Fig. 1:**
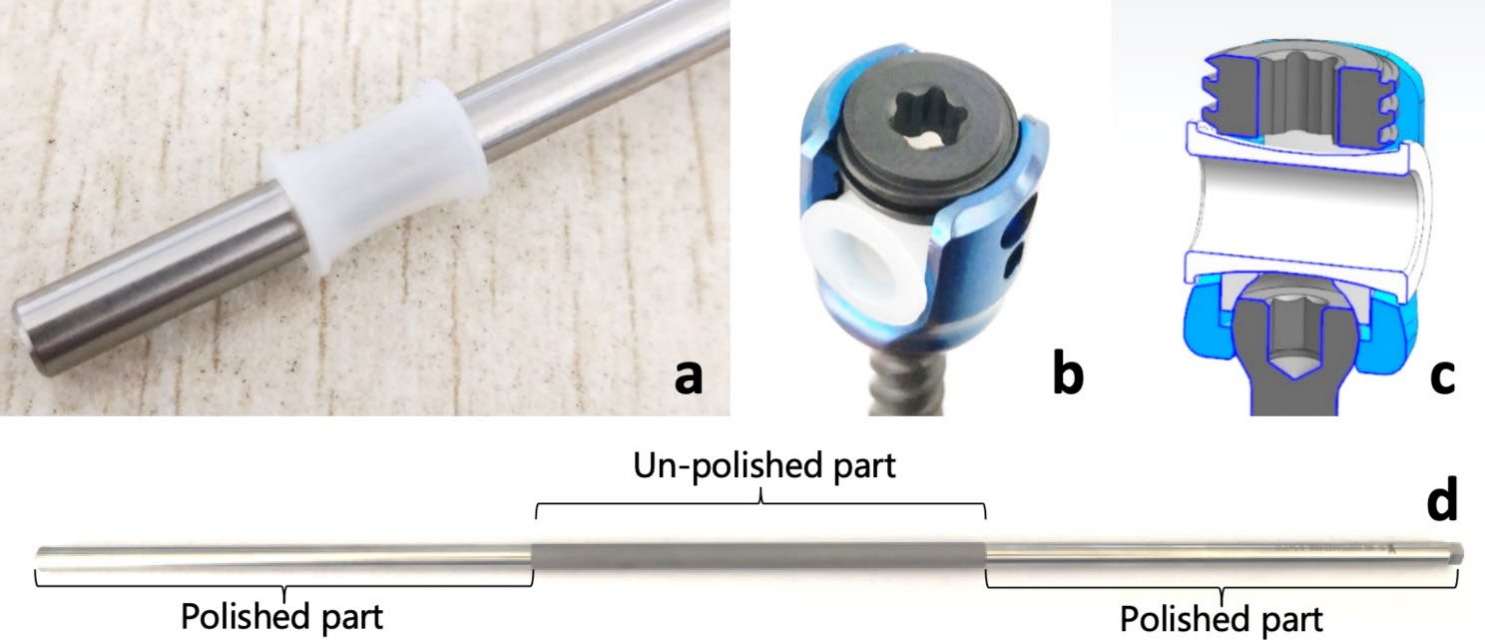
The design of the novel growth guidance system. Figure 1a shows the UHMWPE gasket. Figure 1b shows the UHMWPE gasket can be perfectly fitted into the tulip of the sliding screw and locked by the nut. Figure 1c shows the longitudinal section of the sliding screw with gasket assembled. Figure 1d shows the rod, the sliding part of the rod was polished, the middle part of the rod was un-polished

The Novel Growth Guidance System was designed with titanium alloy Ti-6Al-4 V 4.5-mm diameter rods or 5.5-mm diameter rods, fixed-head screws and poly-axial screws. We made two main modifications to the traditional Shilla system. The first modification, which is also the highlight of the design, we put two ultra-high molecular weight polyethylene (UHMWP) gaskets on the sliding parts of the rod. The UHMWPE gaskets is thin in the middle and thick on the sides, which can be perfectly fitted into the tulip of the sliding screw and locked by the nut (Fig. 1). The outer diameter of the two ends is bigger than the inner distance of the tulip of the screw, in order to avoid the gasket sliding out of the screw. This design may avoid direct friction between the screw and rod, not only to avoid metal debris but also to reduce the sliding friction forces.

Second, we polished the surface of the sliding parts of the rod, to reduce the friction force between the sliding screws and the rods (Fig. 1). The un-polished part of the rods is locked by the fixed screws with the nuts, which are the screws placed at the apex vertebrae. The polished parts of the rod is designed to be captured by the sliding screws, which are placed at the two ends of curve, to decrease the friction forces for better growing of the spine.

### In vitro fatigue test

**Fig. 2:**
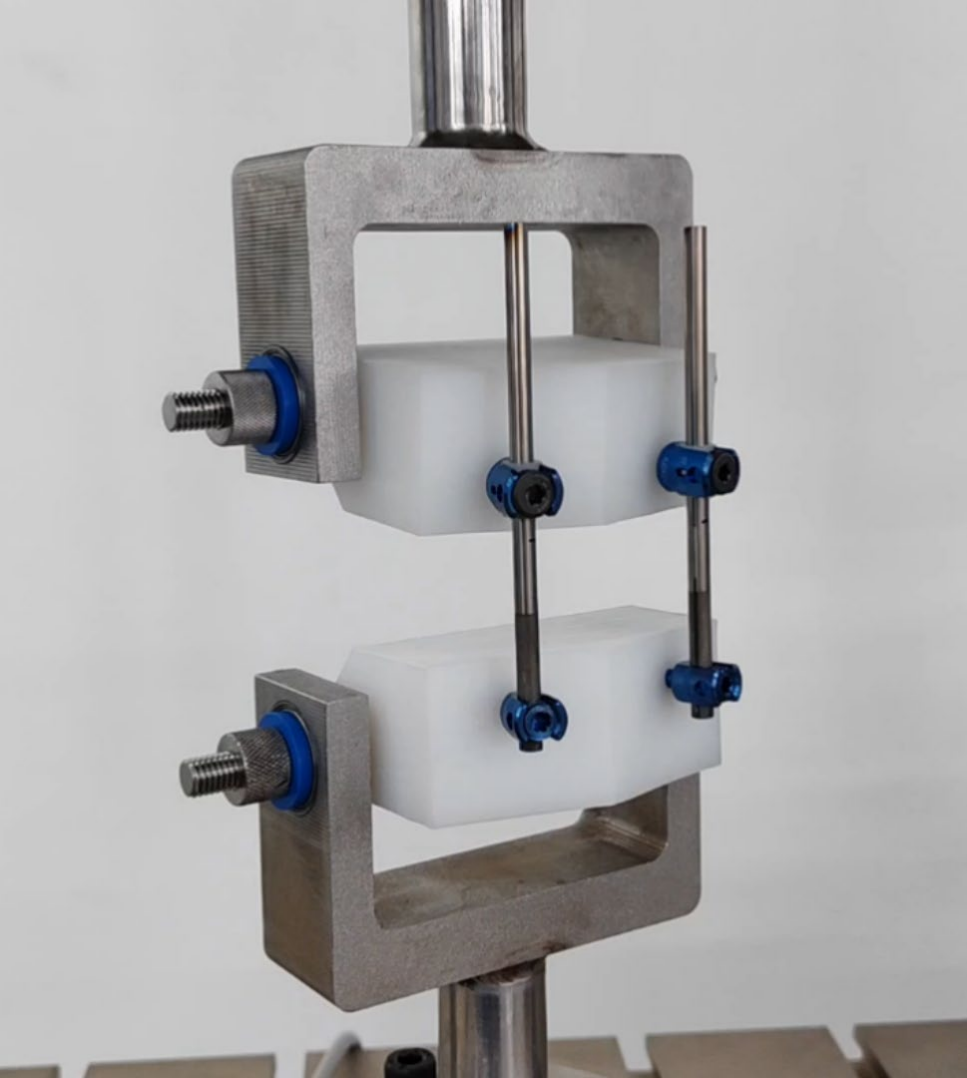
The sample of the novel growth guidance system installed on the MTS test system. The novel growth guidance system (4.5-mm system) was installed on the simulated vertebrae (UHMWPE module) and tested by MTS test system. The sliding poly-axial pedicle screws were placed on the upper simulated vertebra and captured polished parts of the rods with the UHMWPE gaskets. The fixed-head pedicle screws were placed on the lower simulated vertebra and locked un-polished part of the rods

In order to ensure that the UHMWPE gaskets do not experience significant wear and tear in vivo, we conducted an in vitro fatigue test by using the MTS testing system. The novel growth guidance system(4.5-mm system) was installed on the simulated vertebrae (UHMWPE module) and tested by MTS test system. The sliding poly-axial pedicle screws were placed on the upper simulated vertebra and captured polished parts of the rods with the UHMWPE gaskets. The fixed-head pedicle screws were placed on the lower simulated vertebra and locked un-polished part of the rods (Fig. 2). The simulated vertebrae were spaced 30 mm apart initially. The MTS testing system applied dynamic compressive displacement in a sinusoidal waveform to the samples, with a constant displacement of 10 mm and a frequency of 5 Hz, totaling 10 million cycles, roughly equivalent to 10 year of walking in a child [6]. Pre and post-testing involved weighing the UHMWPE gaskets using analytical scales and observing their wear conditions. Three sets of samples were tested, resulting in a total of six UHMWPE gaskets being weighed and observed.

### In vitro sliding displacement test

**Fig. 3:**
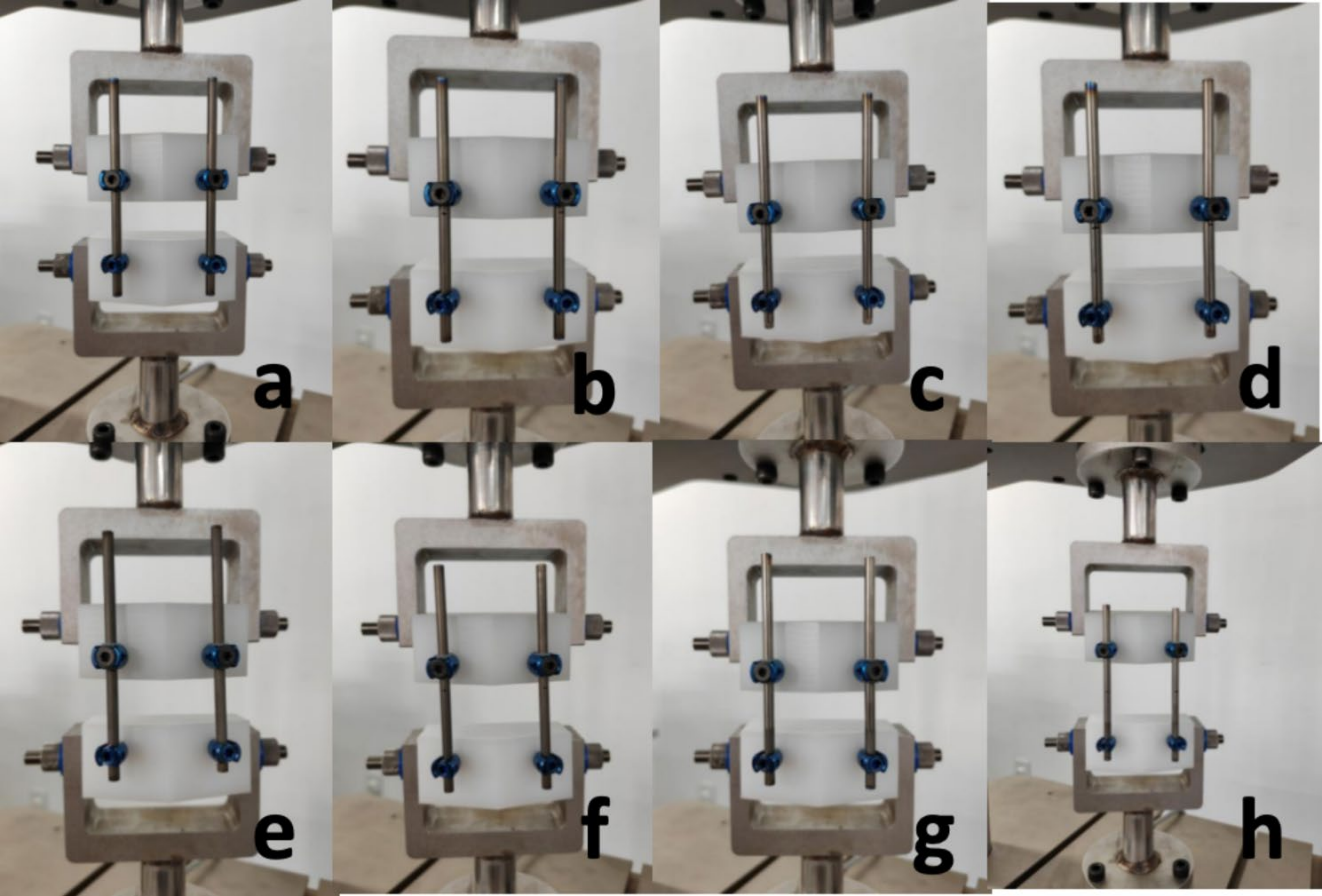
Four groups of samples were installed on the MTS test system. Figure 3a and b are the pre- and post-test of Group A, which is the sliding screw capture un-polished rod without UHMWPE gaskets. Figure 3c and d are the pre- and post-test of Group B, which is the sliding screw capture with polished rod without UHMWPE gaskets. Figure 3e and f are the pre- and post-test of Group C, which is the sliding screw capture un-polished rod with UHMWPE gaskets. Figure 3g and h are the pre- and post-test of Group D, sliding screw capture polished rod with UHMWPE gaskets

To further test the sliding ability of the novel growth guidance system, we conducted an in vitro sliding displacement test by using the MTS testing system. We set 4 groups, 5 samples in each groups: Sliding screw capture un-polished rod without UHMWPE gaskets(Group A), sliding screw capture with polished rod without UHMWPE gaskets(Group B), sliding screw capture un-polished rod with UHMWPE gaskets(Group C), sliding screw capture polished rod with UHMWPE gaskets (Group D) (Fig. 3). All the groups of the implants were installed on the simulated vertebrae same as the way of fatigue test. The MTS testing system applies dynamic compressive loads to the samples in the form of a sinusoidal waveform, with a maximum load of 50 N, a minimum of 5 N, and a frequency of 5 Hz, totaling 300 cycles. This pattern of the motion can mimic the motion of the Shilla system in vivo. After the tests, the maximum sliding displacement of the sliding screws was measured.

### Statistical analysis

Statistical analysis was performed using GraphPad Prism8 (version 8.0.2, GraphPad Software, California, USA). Data were shown as mean ± standard deviation (S.D.). Difference among three or more groups was measured by two-way analysis of variance (ANOVA). Statistical significance was considered at *P* < 0.05.

## Results

### In vitro fatigue test

**Fig. 4:**
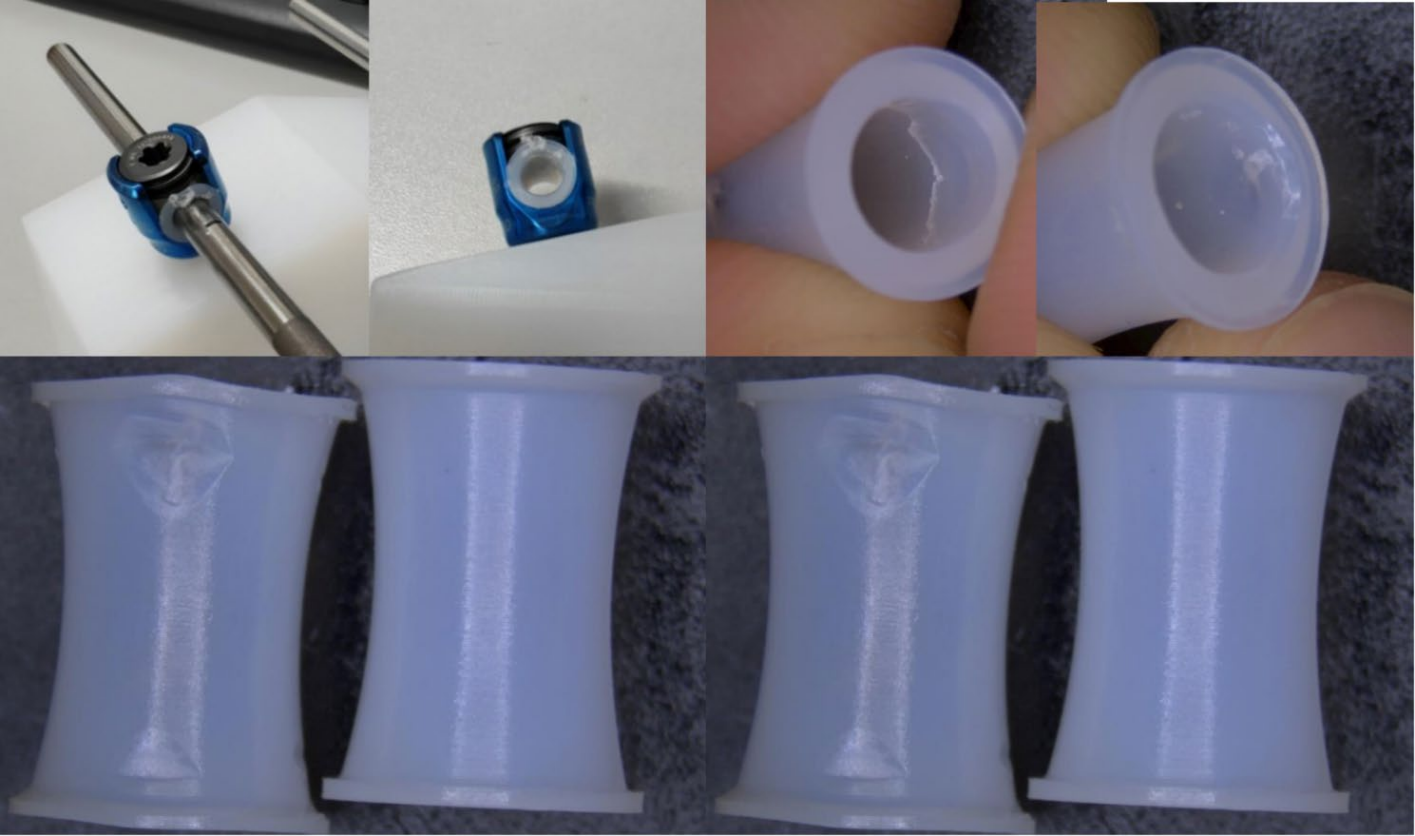
The UHMWPE gaskets samples after the fatigue test. The UHMWPE gaskets samples showed some of the fretting after the fatigue test, but its still isolated and avoided the friction between the screws and rods

After the 10 million cycle of the test, all the UHMWPE gaskets samples showed some of the fretting on the edge of the inner sides, but its still isolated and avoided the friction between the screws and rods (Fig. 4). There was no production of metallic fretting around the sliding screws and rods. The average wear mass of the UHMWPE gaskets was 0.002 ± 0.001 g, less than 1.7% of the original mass (Table 1).

**Table 1:** The results of the fatigue test

	Pre-test mass(g)	Post-test mass(g)	Wear mass(g)
Group 1	0.129	0.127	0.002
	0.129	0.126	0.003
Group 2	0.128	0.126	0.002
	0.128	0.127	0.001
Group 3	0.125	0.124	0.001
	0.125	0.121	0.004
Mean	0.127	0.125	0.002
Standard Deviation	0.002	0.002	0.001

### In vitro sliding displacement test

**Fig. 5:**
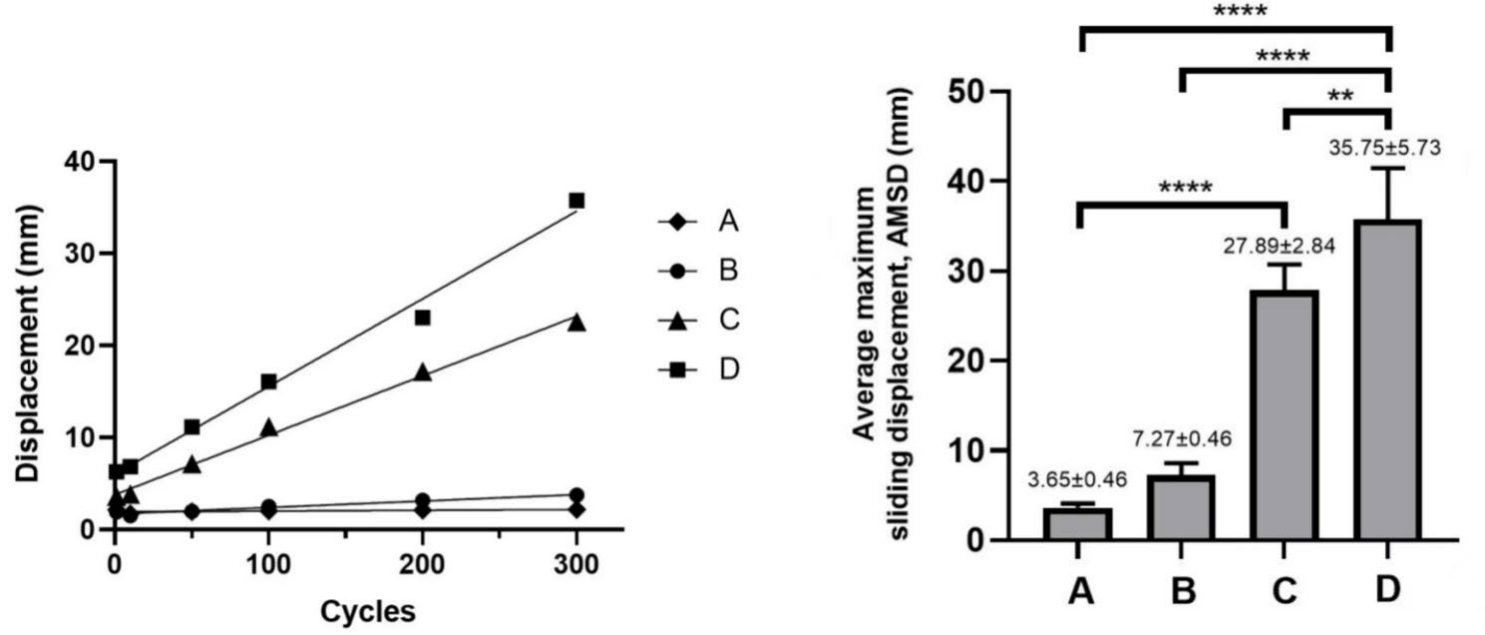
The sliding distance curve and the average maximum sliding displacement of four groups. The left diagram is the sliding distance curve. The x-axis indicated cycles, the y-axis indicated the sliding displacement. The right diagram shows the average maximum sliding displacement of four groups. ****P < 0.0001, **P = 0.0095

The MTS test system can automatically measure the maximum sliding displacement in real time. Figure 5 shows the sliding displacement-cycle curves for the four groups of samples. The group A, the sliding screw capture un-polished rod without UHMWPE gaskets, represented the traditional Shilla system. The average maximum sliding displacement(AMSD) of Group A was 3.65 ± 0.46 mm. The group B, the sliding screw capture polished rod without UHMWPE gaskets, represented changing the rods surface of the traditional Shilla system. The AMSD of Group B was 7.27 ± 0.46 mm. The group C, the sliding screw capture un-polished rod with UHMWPE gaskets. The AMSD of Group C was 27.89 ± 2.84 mm. The The group D, the sliding screw capture polished rod with UHMWPE gaskets, represented the Novel Growth Guidance System. The AMSD of Group D was 35.75 ± 5.73 mm. Through two-way ANOVA, we found that there was no significant difference in the AMSD between Group A and Group B (*P* = 0.4666), but from the data and curves, it became apparent that Group B exhibited AMSD twice as large as that of Group A. Therefore, it can be inferred that polishing the surface of the rod can reduce sliding friction, promoting the relative sliding between the sliding screw and rod. The AMSD of Group C was significantly greater than that of Group A (*P* < 0.0001), and the AMSD of Group D was significantly greater than that of Group B (*P* < 0.0001), indicating that regardless of whether the rod surface been polished, the use of UHMWPE gaskets can reduce sliding friction and promote the relative sliding between the sliding screw and rod. Among the four groups, the AMSD of Group D was significantly greater than that of the other three groups (Group D/Group A *P* < 0.0001, Group D/Group B *P* < 0.0001, Group D/Group C *P* = 0.0095), indicating that the Novel Growth Guidance System had the best sliding performance (Fig. 5).

## Discussion

Growth guidance system was developed from the technique of Luque Trolley. Initially, the Luque Trolley technique employed sublaminar wires and stainless steel rods to correct scoliosis and allow for spinal growth [13]. However, the implantation of sublaminar wires could strip the peritoneal of spine, caused interlaminar ankylosis and eventually autofusion [14]. Additionally, sublaminar wires had limit corrective forces over the vertebra. With the advancement of pedicle screw technology and spinal correction techniques, particularly vertebral derotation techniques, McCarthy improved the Luque Trolley technique and invented the Shilla technique. Shilla technique corrected the apex of the scoliosis maximally with osteotomies and vertebra derotation, by using the fixed-head pedicle screw. At the two ends of the scoliosis, the Shilla screws were placed to allow the spine growing in a normal alignment with the inherent growth potential of the spine. The animal experiment result supported the use of the Shilla system in humans by allowing for continued guided growth. Subsequent clinical studies have confirmed that the Shilla technique can be widely used to treat various types of early-onset scoliosis, with a main curve correction rate reaching nearly 50% [7, 8]. There was also significant growth observed in the height of T1-T12, T1-S1, and space available for the lung [15, 16]. Meanwhile, throughout the entire treatment, patients treated with the Shilla technique undergo significantly fewer surgeries on average compared to those treated with the traditional growing rods. Additionally, the average treatment cost for patients treated with the Shilla technique was lower than for those treated with traditional growing rods or magnetically controlled growing rods [16, 17].

However, the Shilla technique was primarily criticized for two major drawbacks, metal debris and the weak ability of the growth promotion [18]. The metal debris was created by the sliding between the screws and rods, which may increase the concentration of metal ions in local tissues and blood. Actually, in the goat experiment, metallic wear debris was observed in the soft tissue and lymph nodes adjacent the Shilla screws [6]. The metallic tissue staining was also observed in human patients population [19]. In a clinical study, Lukina tested the content of Ti, AL and V metal ions in whole bloods and local tissues around the sliding instruments, found that the Ti and V ions in blood increased 2.8 and 4 time respectively, Ti ions in local tissues was more than 1500-fold higher than the control group [12]. Metallic debris also can induce a large inflammatory response of the macrophages [11]. Our novel growth guidance system addressed this issue by altering the friction interface between the screws and the rods to reduce metal debris. The UHMWPE gaskets fitted onto the rod can be perfectly positioned within the tulip of the screw, thus preventing direct metal-to-metal contact between the sliding screws and the rods. We chose UHMWP as the material for the gaskets because it was a highly biocompatible polymer with excellent wear resistance. It had been widely used in orthopedic and spinal surgery implants, such as artificial discs, sublaminar wires, and artificial joints [20]. Fatigue tests confirmed that after 10 million cycles, the wear of UHMWPE gaskets was minimal, and they still effectively prevented direct contact between the screws and the rods. Therefore, we believed that the application of UHMWPE gaskets was an excellent method for improving the friction interface and avoiding metal debris.

The ability of the growth promotion was the second concern about the technique. According to the study conducted by the inventor’s institution, the Shilla patients had less T1-S1 height increase compare to the traditional growing rods [15]. The study outside the inventor’s institution, showed that EOS patients treated with Shilla technique was approxiamately 1/3rd of predicted normal T1-S1 growth, less than 1/3^rd^ of growth reported in the inventor’s institution [10]. The primary reasons for the limited growth-promoting capability of the Shilla technique are twofold: firstly, it lacks of external distraction force, and secondly, excessive friction between screws and rods restricts spinal growth. Based on the traditional Shilla technique, we improved the friction interface between screws and rods by applying UHMWPE gaskets and polishing the rod, to reduce the friction, facilitate screw sliding and minimize restriction on spinal growth. From our experimental results, it was evident that merely by polishing the sliding part of the rod surface can facilitate the sliding of the screws. Additionally, the use of UHMWPE gaskets significantly enhanced screw sliding.

Although the novel growth guidance system is a modification to the Shilla system, we hope our approaches to change the interface of sliding instruments can be also applicable to the all growth friendly techniques involved the sliding elements. Instead of using Shilla sliding screws, Agarwal modified the Shilla technique by using dominos as a sliding elements [21–23]. Cody Bunger(CB) technique combined a single concave MCGR with a sliding rod on the convex side to control the apex, which also utilized dominos as sliding elements [24, 25]. Same combination applied in the spring distraction system also [21]. An fitted size UHMWP gasket can also be inserted into the holes of the domino. Additionally, the sliding part of the rod can be polished to minimize metal debris generation and decrease frictional forces.

This study is only a preliminary in vitro experiment by using the MTS system, which is the main limitation of the study. The efficacy of the system, including the metallic and UHMWPE debris created by the system, and the sliding ability, should be assessed in animal model in the future. Also in the future, we believe that the novel growth guidance system can be applied in human with a bright future.

## Conclusion

In conclusion, we modified the Shilla technique and designed a novel growth guidance system. Two major modifications were made to the traditional Shilla system, including the use of UHMWPE gaskets to avoid direct contact between the screw and rod, and polishing the surface of the sliding part of the rod, which may significantly reduce the metallic debris and promote spine growth. The fatigue test and sliding dislocation test demonstrated the durability and better glidability of the system. An in vivo animal experiment should be performed to further verify the system.

## Data Availability

The datasets used and/or analysed during the current study are available from the corresponding author on reasonable request.
